# Empowering Patients With Functional Neurological Disorders (FND) Through Information to Facilitate Informed Decision-Making and Active Condition Management

**DOI:** 10.1192/bjo.2024.442

**Published:** 2024-08-01

**Authors:** Soogeun Lee, Bruce Tamilson

**Affiliations:** South West London and St George's Mental Health NHS Trust, London, United Kingdom

## Abstract

**Aims:**

This service improvement project seeks to empower individuals diagnosed with Functional Neurological Disorders (FND) by delivering comprehensive information, facilitating informed choices about their care, and encouraging an active role in managing their health.

**Methods:**

Information was gathered relating the concerns and expectations of FND patients upon receiving a diagnosis or attending the neuropsychiatric clinic at a regional neuroscience centre. The identification of a patient information leaflet as a valuable resource became apparent. Consequently, a meticulously designed leaflet was developed to educate patients about their condition, providing useful tips and resources. The content of the leaflets underwent a thorough series of reviews, incorporating input from various professionals within the multidisciplinary team, with additional consideration given to feedback from service users. To assess the impact of this intervention, feedback is required from both clinicians and end-users.

**Results:**

The patient information leaflet contains information designed to enlighten patients about their condition, incorporating psychoeducational content on self-help strategies and available treatment modalities. It also highlights support resources available to them. The leaflet can be conveniently stored in the neurology and neuropsychiatric clinic areas for easy clinician access and distribution to relevant patients. Additionally, it is available in PDF format, enabling clinicians to print it in satellite clinics, and medical secretaries can email it to patients along with clinic letters as directed by the clinicians. Initial feedback from patients and clinicians has been overwhelmingly positive, with many considering it an essential intervention.

**Conclusion:**

This service improvement, realized through a relatively modest intervention, can lead to a substantial impact on patient care and satisfaction. Providing patients with pertinent information is crucial for fostering informed decision-making and empowering them to take an active role in their care. Especially for conditions historically stigmatized and misunderstood, it is imperative to disseminate up-to-date information, establishing a reliable and endorsed source to dispel stigma for both patients and their families.

